# The Main Drivers of Wetland Changes in the Beijing-Tianjin-Hebei Region

**DOI:** 10.3390/ijerph16142619

**Published:** 2019-07-23

**Authors:** Liyun Zhang, Quan Zhen, Min Cheng, Zhiyun Ouyang

**Affiliations:** 1 State Key Laboratory of Urban and Regional Ecology, Research Center for Eco-Environmental Sciences, Chinese Academy of Sciences, Beijing 100085, China; 2 College of Resources and Environment, University of Chinese Academy of Sciences, Beijing 100049, China; 3 School of Public Health, Bengbu Medcial College, Bengbu 233030, China

**Keywords:** Wetlands conservation, land-use and land-cover (LULC), artificialization, agriculture production, aquaculture production

## Abstract

Wetlands are the most threatened ecosystem in China, and wetland conservation is a national priority because of their importance for water security, flood mitigation, and biodiversity conservation. A goal has been established for the Beijing-Tianjin-Hebei Region (BTH) to recover 340 km^2^ of wetlands by 2020. To guide restoration and protection efforts, policymakers need information on the trends of wetland loss, conversion of wetlands, and their associated human drivers. The main drivers of changes in different wetland types in the BTH were identified and quantified from 2000 to 2015. In 2015, there was 6264.07 km^2^ less wetland area than in 2000, with the remaining wetlands primarily located in Hebei and Tianjin. Reservoirs/ponds were the most abundant wetland type, followed by herbaceous swamps, rivers, canals and channels, and then lakes as the least represented. There were continuous losses of wetlands from 2000 to 2015, with marked decreases for rivers (30.48%), channels/canals (23.30%), and herbaceous swamps (16.12%). However, there was an increase in the area of lakes and reservoirs/ponds, with increases of 54.96% and 3.47%, respectively. The largest changes in natural wetlands were due to agricultural production followed by artificialization and grassland expansion. The driving forces of the observed changes were specific to each local region. According to an aggregated boosted trees (ABT) analysis, gross farm production, total aquatic products, and irrigated area were the top three drivers of the decrease in natural wetlands, which agreed with the main patterns of change in the BTH. The purpose of this study was to provide guidance for policy makers working to meet the 2020 BTH wetland recovery target. Recommendations were provided at the provincial level, including water transfers across provincial boundaries, the control of agricultural expansion, exploration of species-specific irrigation deficits, a reduction in the artificialization of land surfaces, the development of a sustainable intensified aquaculture model, and the promotion of awareness of wetland importance among local people.

## 1. Introduction

Wetlands are one of the most productive ecosystems on the planet, supporting millions of people and providing important services, such as pollutant purification, flood control, and supporting biodiversity [1]. Almost 50% of the global wetlands were lost during the 20th century [2]. Patterns of wetland changes are the result of social, economic, technological, and policy issues [3,4], often acting at different scales that can have profound impacts on the livelihood of people, biodiversity, and the provisioning of ecosystem services. The wetland area in China decreased by 33% from 1978 to 2008. The transformation of wetlands varied, with natural wetlands being transformed into no-wetland land use types before 1990 and into artificial wetlands after 1990 [5]. As a developing country, China relies heavily on wetland ecosystems and the services they provide [6], while wetland degradation and loss may deteriorate China’s ecosystem security. For example, the massive declines in lake area along the Yangtze River in the late 1980s due to land reclamation has contributed to severe flooding, which has led to the loss of thousands of human lives and property damage costing approximately $36 billion USD [7]. The Chinese government is making substantial investments in wetland conservation [8]. Despite these government efforts, anthropogenic drivers continue to perpetuate wetland losses. There is a pressing need for a deeper understanding of the human impact on wetland changes.

The majority of studies in this field have focused on the drivers of wetland changes [1,9,10,11] and wetlands are often considered a land-cover class (e.g., non-urban land-use) [12]. Furthermore, these studies have typically not evaluated the conversion of wetlands to other land-use types, and the assessment of the drivers has been qualitative. To address this gap, Richards and Friess [13] analyzed the drivers of deforestation on the conversion of mangroves to different land-use types across Southeast Asia, and revealed the drivers of deforestation in a quantitative way. The only drawback was that this study did not examine the conversion of other land-use types to mangroves to assess the status of restoration efforts. Natural wetlands (e.g., rivers, lakes, and swamps) are superior to artificial wetlands in providing ecosystem services and have a greater resilience when suffering from stresses [14], which suggests that there is a need to primarily focus on the recovery of natural wetlands, rather than considering all wetlands as a singular group.

The Beijing-Tianjin-Hebei region (BTH) is one of the most important economic and political centers in China, accounting for 8.11% of the population and 10.12% of the Gross Domestic Product (GDP) in 2015 [15]. Due to its large population, rapid development, and semi-arid environment [16], the BTH faces serious water shortage problems [17]. In 2015, the per capita water availability in the BTH was 124 m^3^ [18,19,20], which is far below the United Nations defined level of absolute water security of 500 m^3^ per capita (World Water Assessment Program 2012 (WWAP)). Furthermore, the BTH suffers from poor water quality across the region. In Beijing, 42% of the total length of rivers is below Grade V (considered not suitable for any human uses), while the corresponding figure in Tianjin is 73% [21]. The Urban Expansion Index for the BTH is 140.66, which was much higher than the other two main urban agglomerations in China—the Yangtze River Delta (100.07) and Pearl River Delta (77.70) [22]. Wetlands are vulnerable and sensitive to land-use and land-cover (LULC) changes [23] and anthropogenic drivers, such as over-exploitation and sea-level rise due to global climate change [24,25]. The National Economic and Social Development plan for the BTH Region during the 13th five-year plan period was the first to be published across provinces, which made it necessary to take wetlands in the three provinces into consideration together.

Much of the work that has been conducted on this topic has been limited to isolated areas [26,27,28,29]. The purpose of this study was to analyze a large and heavily populated geographical area and determine underlying causes for changes to its wetlands. The conversion between wetlands and other land-use and land-cover (LULC) types was evaluated. The Forest and Natural Ecological Protection and Restoration Plan in the BTH, published by State Forestry Bureau and Forestry Bureaus of the three provinces, stressed that the area of wetland restoration and reversion from cultivated land should be 340 km^2^ (510,000 mu) by 2020. Policymakers need information on the patterns of change of different wetland types and the human drivers causing declines in wetland area to guide restoration and protection efforts, and therefore meet the recovery goal for 2020.

In this study, the BTH was examined to analyze the wetland changes and the drivers of these changes from 2000 to 2015 using a remote sensing and geographic information system (GIS) methodology coupled with field data. The objectives of this study were to: (1) analyze the distribution of wetlands, considering the different wetland types in the BTH; (2) quantify changes in the wetland area in the BTH; and (3) identify the spatial and temporal variation of wetlands, and the drivers of change to advise policymakers on future conservation efforts.

## 2. Materials and Methods 

### 2.1. Study Area

**Figure 1:**
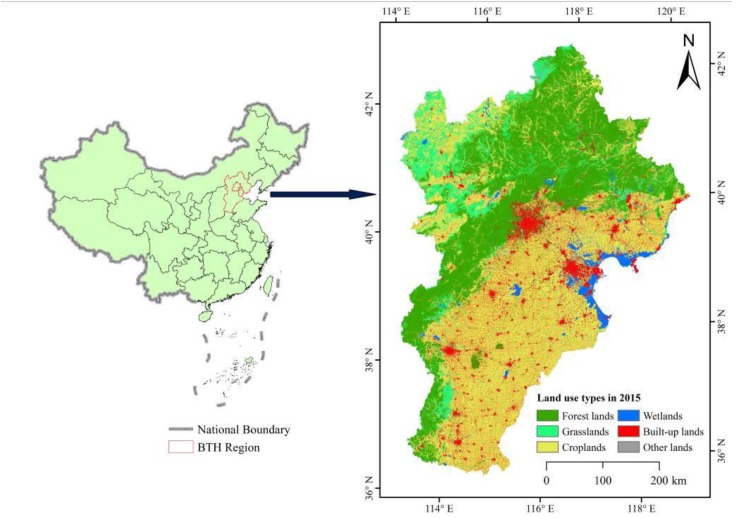
Location of the study site known as the Beijing-Tianjin-Hebei region (BTH).

The BTH has an elevation ranging from −46 to 2823 m and extends over an area of 216,020 km^2^ at respective latitudes and longitudes of 36°01′ N to 42°37′ N and 113°04′ E to 119°53′ E (Figure 1). The BTH is located in the northern portion of the North China Plain and forms part of the Bohai Rim, which is in the transition zone from the Taihang Mountains in the west to the Yanshan Mountains in the north and southeast. The BTH has a high population density of 516 people per km^2^. It is characterized by a sub-humid continental monsoonal climate, with a mean annual precipitation of 543.33 mm. The BTH contains important wetlands, such as Baiyangdian Lake (319 km^2^), Yongding River (47,016 km^2^), Changli Golden Coast Nature Reserve (336 km^2^), and North Dagang Wetland Nature Reserve (442 km^2^). Wetlands only account for 2.90% of the total land area in BTH; however, they play a critical role in maintaining biodiversity and sustaining the production of ecosystem services that are critical for human well-being. 

### 2.2. Wetland Data

Maps of LULC and other relevant data for the BTH were examined for the years of 2000 and 2015. We obtained the necessary LULC data from an official database titled “China’s National Ecosystem Assessment and Ecological Safety” [30], which is overseen by the Research Center for Eco-Environmental Sciences, Chinese Academy of Sciences. The database was compiled using object-oriented classification technology based on remote sensing data from Landsat Thematic Mapper/Enhanced Thematic Mapper (TM/ETM) and Chinese Environmental Disaster Alleviation Satellites(HJ-1) at 30 m resolution [31]. The LULC was divided into eight land classes (forests, bush woods, grasslands, wetlands, deserts, croplands, built-up lands, and others). Wetlands were further divided into three natural subclasses (herbaceous swamps, rivers, and lakes) and two artificial subclasses (reservoirs/ponds and channels/canals). The LULC data in BTH was verified using 4405 field data points, with an overall accuracy of 92.47% for the land classes and 87.18% for the wetland subclasses.

### 2.3. LULC Analysis by Geographic Information System (GIS)

This analysis was built on two high quality LULC datasets for 2000 and 2015. Wetland distribution maps were cross-referenced with LULC maps and the distribution of wetland changes was mapped by pixel using the ArcGIS software. The distribution and conversion of wetlands was specific for each province and geographic region. 

### 2.4. Driving Forces of the Decrease in Natural Wetlands

The forces driving the decrease in natural wetlands can be divided into two groups: factors related to human activities and natural factors. We selected annual precipitation and slope as the primary natural variables. In addition, factors such as gross domestic product (GDP), primary industry, secondary industry, tertiary industry, total population, rural population, urban population, irrigated area, total aquatic products, grain yield, and gross farm production were chosen to explore the effects of socioeconomic development on the decrease of natural wetlands. Aggregated boosted trees (ABT) analysis was carried out using the gbm package with 10,000 trees for the boosting, and 10-fold cross-validation. This model is a machine learning method based on a decision tree, which aims to achieve accurate prediction and explanation [32]. Compared to the traditional methods of linear correlation and multiple linear regression, our model was better at dealing with nonlinearities and interactions, and more importantly, at quantitatively evaluating the relative influence of socioeconomic development and climate factors on natural wetlands decrease (n.trees = 10,000 and cv.folds = 10). Furthermore, we conducted other qualitative analyses on the decrease of natural wetlands, which are detailed in the discussion section. In this manner, we identified and quantified the effects of human activity and natural changes on the decrease of wetlands.

## 3. Results

### 3.1. Changes in Wetland Area and Distribution

**Figure 2:**
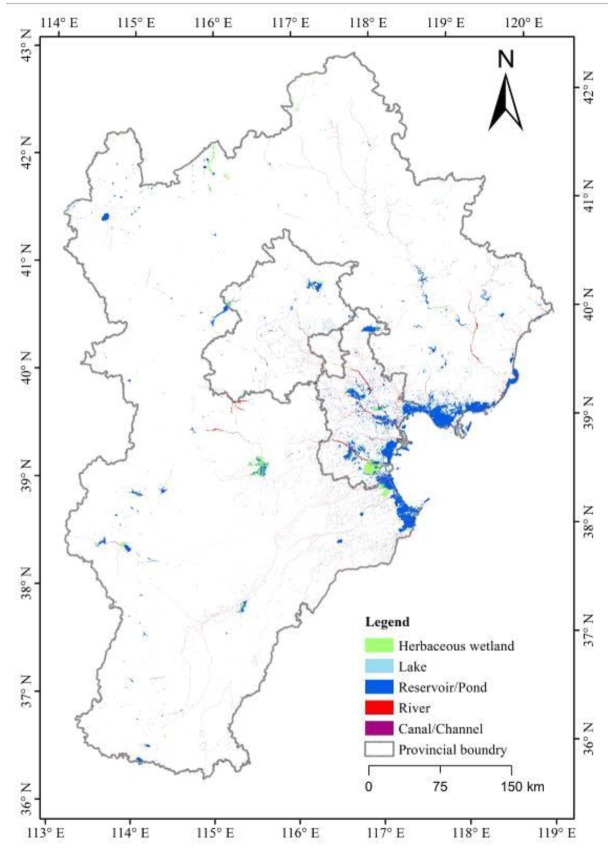
Wetland distribution in the Beijing-Tianjin-Hebei region (BTH) in 2015.

The main type of wetland in the BTH was artificial wetlands, especially reservoirs/ponds, which were mainly located in the Bohai Rim in Hebei and Tianjin provinces (Figure 2). The total area of each wetland subclass was compared between the years 2000 and 2015 (Table 1). Over this period, the wetland area decreased by 5.88% from 6655.49 to 6264.07 km^2^. However, there were unique changes among the wetland types. The area of lakes and reservoirs/ponds increased by 54.96% and 3.47%, respectively, while the area of herbaceous swamps, rivers, and canals/channels decreased by 16.12%, 30.48%, and 23.30%, respectively. In total, the natural wetland area decreased by 22.89% and the artificial wetland area increased by 2.05%.

Wetland distributions and trends for each province were determined. Similar wetland distributions and trends were found for Beijing, Tianjin, and Hebei. The dominant wetland type in all three provinces was reservoirs/ponds, followed by herbaceous swamps and rivers. The area of lakes and canals/channels was proportionally small, accounting for less than 5% of the total wetland area. The wetland area, including natural wetlands, in the three provinces decreased from 2000 to 2015. In Beijing, the area of herbaceous swamps increased, while the area of all other of wetland types decreased. In Hebei, the natural wetland area decreased by 24.99%, although the lake area increased by 12.99 km^2^. The area of reservoirs/ponds increased by 364.96 km^2^, while the area of canals/channels decreased by 46.71 km^2^. In Tianjin, the natural and artificial wetland areas decreased by 17.53% and 5.95%, respectively, and the only type of wetland to increase was rivers.

**Figure 3:**
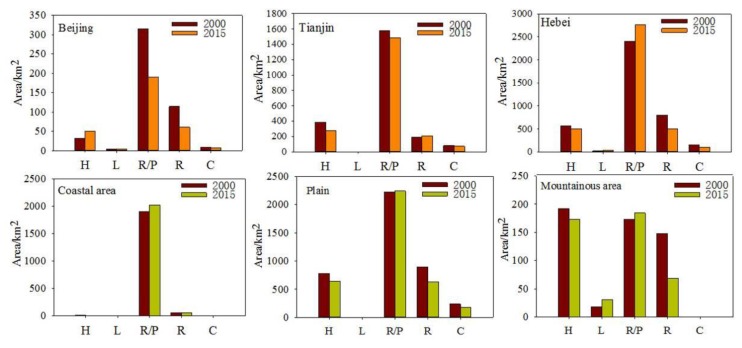
Comparison of total wetland area for each wetland subclass in 2010 and 2015 for Beijing, Hebei, Tianjin, coastal areas, plains, and mountainous areas. Note: H = herbaceous swamps; L = lakes; R/P = reservoirs/ponds; R = rivers, C = canals/channels.

To determine how the wetland distribution and trends varied geographically, the BTH was subdivided into coastal areas, plains, and mountainous areas. The aim was to compare whether geographical variations of wetlands mirrored administrative variations of wetlands and to address any mismatches between environmental boundaries and social boundaries. Coastal areas were defined as regions having a coastline and extended up to 10 km inland. The designations for plains and mountainous areas were assigned according to altitude. Coastal regions were excluded from this assignment. Regions with altitudes less than 500 m were defined as plains and regions with altitudes greater than 500 m were defined as mountainous areas. Wetlands were mainly distributed in the plains (59.20%), followed by coastal areas (33.49%) and mountainous areas (7.32%). Reservoirs and /ponds were the dominant wetland type in coastal areas (96.32%) and plains (60.55%) in 2015, and herbaceous swamps and reservoirs/ponds were main ones in mountainous areas (Figure 3). The natural wetland area in the three areas experienced a continuous decline, while the artificial wetland area increased in coastal and mountainous areas. In terms of specific wetland types, the area of herbaceous swamps and reservoirs/ponds decreased in all three areas, while the area of lakes decreased in plains and increased in mountainous areas. The area of rivers and canals/channels increased in coastal areas, but decreased elsewhere.

### 3.2. Wetland Changes

Wetland transformations were characterized in three ways from 2000 to 2015: wetlands-in (other land-use types converted into wetlands), wetlands-out (wetlands converted into other land-use types), and wetlands-internal (conversion between wetlands).

**Figure 4:**
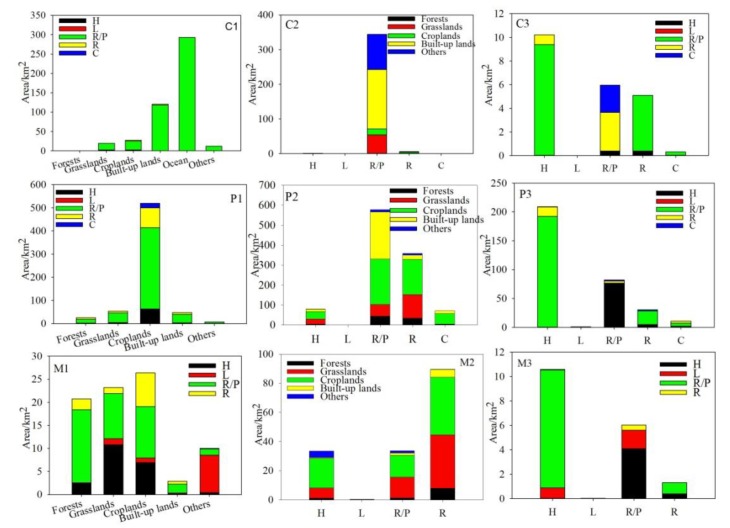
Modes of spatial wetland transformation. Note: C = coastal areas; P = plains; M = mountainous areas; 1 = wetlands-in (other land-use types converted into wetlands); 2 = wetlands-out (wetlands converted into other land-use types); and 3 = wetlands-internal (conversion between wetlands).

The total area of wetlands-in was 1208.56 km^2^, with coastal areas, plains, and mountainous areas accounting for 39.15%, 53.98%, and 6.88%, respectively (Figure 4). In coastal areas, 462.77k m^2^ of other land use types were converted into reservoirs/ponds, with reclamation (ocean converted into reservoirs/ponds) accounting for 63.20%. There was only 9.87 km^2^ of other land use types that were changed into natural wetlands. In plains, there were 473.58 and 178.74 km^2^ of other land use types (mainly cropland) converted to artificial and natural wetlands, respectively. In mountainous areas, the area of other land types transformed to natural and artificial wetlands was almost the same in percentage terms. Croplands, grasslands, and forests were the main land use types converted to wetlands. There was a relatively similar pattern of wetlands-in conversion in all three provinces. Croplands was the main land use type converted into wetlands in all provinces, and was mainly transformed into reservoirs/ponds. Beijing only accounted for 4.03% of wetlands-in area, with the main transformation being from croplands to reservoirs/ponds and herbaceous swamps, which accounted for 33.10% and 20.78%, respectively. Hebei accounted for most of the region’s wetlands-in conversion. The most important end-point of the transformation was reservoirs/ponds (667.05 km^2^), followed by rivers (108.19 km^2^) and herbaceous swamps (76.57 km^2^). After cropland (415.00 km^2^), the ocean (191.08 km^2^) was the next most important source of land for conversion. In Tianjin, the transformation to reservoirs/ponds accounted for 93.97% of the area of wetlands-in conversion and the transformation to reservoirs/ponds occurred mainly from croplands (114.27 km^2^) and the ocean (101.40 km^2^).

The total area of wetlands-out was 1,599.12 km^2^_,_ with coastal areas, plains, and mountainous areas accounting for 21.99%, 68.20%, and 9.81%, respectively. In coastal areas, 344.35 km^2^ of reservoirs/ponds were converted to other land uses (Figure 4). In plains, there were 493.75 and 84.66 km^2^ of wetlands converted into croplands and built-up lands, which accounted for 45.27% and 26.10% of the total wetlands-out conversion, respectively. The main transformations were reservoirs/ponds to croplands and built-up lands at 227.76 and 236.99 km^2^, respectively. A total of 359.33 km^2^ of rivers were changed to other land uses, mainly cropland (176.93 km^2^) and grassland (117.18 km^2^). In mountainous areas, 74.90 km^2^ of wetlands were converted to cropland, while 58.20 km^2^ were converted to grassland. The main source was rivers, which accounted for 57.09% of the wetlands-out converted area. In Beijing, there were no clear trends in the transformations. Conversion into grassland and cropland were the two main transformations (accounting for 62.74 and 56.27 km^2^ of the area converted, respectively). The transformation was mainly from reservoirs/ponds (136.50 km^2^) and rivers (59.52 km^2^). In Hebei, 422.64 km^2^ of wetland was converted to cropland, which was followed by grassland (217.27 km^2^) and built-up land (185.84 km^2^). Rivers (390.03 km^2^) and reservoirs/ponds (375.08 km^2^) were the dominant land use types that were converted. In Tianjin, the largest transformation was from reservoirs/ponds to built-up land, which accounted for 47.19% of the total area converted. Another important end-point of these conversions was cropland, which experienced an expansion of 110.57 km^2^. 

**Figure 5:**
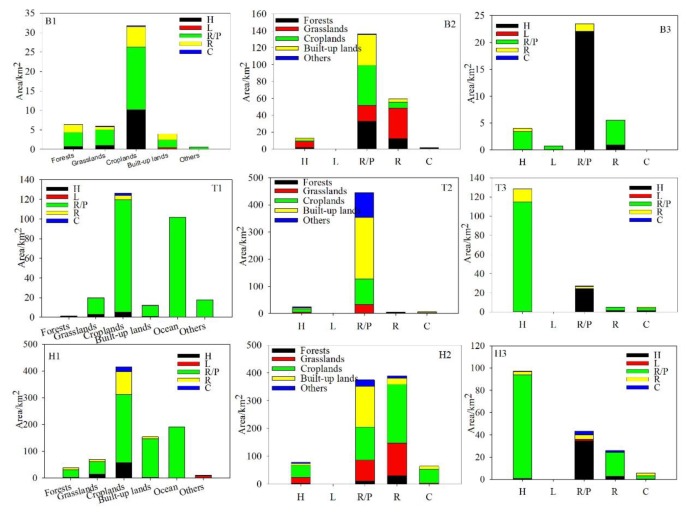
Modes of spatial wetland transformation. Note: B = Beijing; H = Hebei; T = Tianjin; 1 = wetlands-in (other land-use types converted into wetlands); 2 = wetlands-out (wetlands converted into other land-use types); and 3 = wetlands-internal (conversion between wetlands).

The total area of wetland-internal was 371.78 km^2^, which mainly occurred in the plains (89.36%), followed by coastal areas (5.81%) and mountainous areas (4.83%). More than half of the transformation was accounted for by natural wetlands converting to artificial wetlands in three geographical areas. The transformation from herbaceous swamps to reservoirs/ponds was the dominant change, accounting for an area of 9.40, 192.34, and 9.65 km^2^ for coastal areas, plains, and mountainous areas, respectively. Transformations from reservoirs/ponds or rivers were considerable, especially in the plains (Figure 4). In all provinces, herbaceous swamps and reservoirs/ponds were the two active wetland types that were most commonly changed, while rivers were also commonly changed in Beijing and Heibei. In Beijing, 23.45 km^2^ of artificial wetlands was changed to natural wetlands. The transformation from reservoirs/ponds to herbaceous swamps occurred over an area of 22.07 km^2^. In Hebei and Tianjin, the transformation from natural wetlands to artificial wetlands was the dominant change. The main transformation was from herbaceous swamps to reservoirs/ponds (Figure 5).

### 3.3. Natural Wetlands Patterns of Change from 2000 to 2015

There is a considerable amount of published literature that documents the superior ecosystem services that natural wetlands provide over artificial wetlands. In light of this, changes in different types of natural wetland were used to quantify the key proximate drivers of change. Six main change patterns were identified as the causes of the decrease in natural wetlands: urbanization (wetlands converted into built-up lands), agriculture (wetlands converted into cropland), artificialization (natural wetlands converted into artificial wetlands), internal conversion between natural wetlands, and grassland expansion (wetlands converted into grassland), with minor drivers lumped into an “others” category. 

In BTH, the main changes were agriculture, artificialization, and grassland expansion, which accounted for 33.16%, 29.06%, and 22.46% of the converted area, respectively. Artificialization was the dominant factor for the decrease in the area of herbaceous swamps and lakes. Agriculture and grassland expansion were the dominant drivers for the decrease in the area of rivers (Table 2 and Table 3).

The driving forces varied with the location and wetland type. In coastal areas, the natural wetland area decreased by 351.65 km^2^, with the contribution of the different driving forces being, from largest to smallest, artificialization, agriculture, urbanization, internal conversion, grassland expansion, and others. Decreases in the area of herbaceous swamps and rivers were due to the same dominant factor of artificialization, but the second most important factor was different, being internal conversation for herbaceous swamps and agriculture for rivers. In plains, the area of natural wetlands decreased by 1090.57 km^2^ and the ranking of the driving forces, from largest to smallest, was artificialization, agriculture, grassland expansion, others, urbanization, and internal conversion. For all three wetland types artificialization was the dominant factor in their decrease, but for rivers, agriculture and then grassland expansion were also important factors. In mountainous areas, the natural wetland area decreased by 156.90 km^2^ and the ranking of the driving forces, from largest to smallest, was agriculture, grassland expansion, others, artificialization, urbanization, and internal conversion. For herbaceous swamps, agriculture was the dominant factor in their decrease, followed by artificialization. For lakes, others was the dominant factor, followed by grassland expansion and agriculture, while grassland expansion was the main driver of the decrease in area of rivers (Table 2).

In Beijing, the natural wetland area decreased by 210.96 km^2^, with the contribution of different driving forces being, from largest to smallest, grassland expansion, others, artificialization, agriculture, urbanization, and internal conversion. For herbaceous swamps, grassland expansion and artificialization were two main factors in order, while for lakes artificialization was the dominant factor and grassland expansion was the most important factor for rivers. In Hebei, the natural wetland area decreased by 908.12 km^2^, with the contribution of different driving forces being, from largest to smallest, agriculture, grassland expansion, artificialization, others, urbanization, and internal conversion. The three different wetland types had different dominant factors, artificialization, others, and agriculture for the decrease in area of herbaceous swamps, lakes, and rivers, respectively. In Tianjin, the natural wetlands decreased by 480.20 km^2^, with the contribution of different driving forces being, from largest to smallest, artificialization, internal conversion, agriculture, urbanization, grassland expansion, and others. The dominant factor in the decrease in area of herbaceous swamps and rivers was artificialization, but internal conversation, agriculture, and urbanization played almost the same roles after artificialization in the decrease in area of rivers (Table 3).

### 3.4. Drivers of the Decrease in Natural Wetland Area

**Figure 6:**
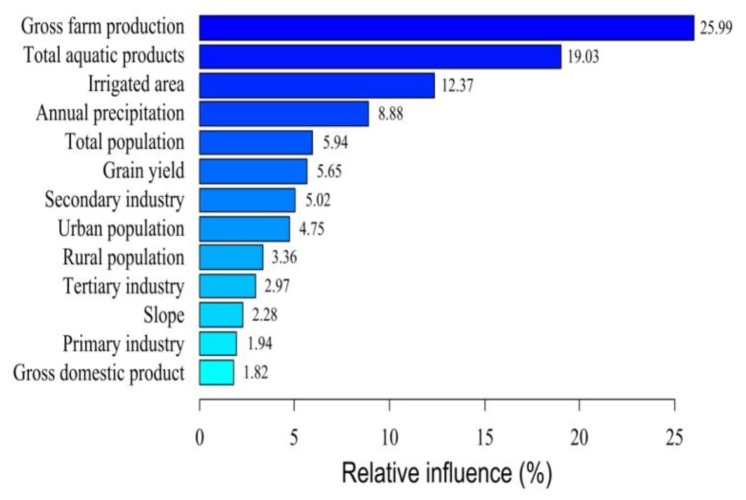
Relative influence of socioeconomic and natural factors on the decrease of natural wetland area.

Drivers of natural wetlands decrease were analyzed in a statistic way. Across the BTH, the ABT analysis and pattern of wetlands change were in broad agreement (Figure 6). According to the ABT analysis, gross farm production had the largest relative influence (25.99%) on natural wetland area, while irrigated area (12.37%) took the third order, which agreed with the pattern of change in agriculture. Total aquatic products (19.03%), which took the second order, had strong connections with artificialization. The first three independent variables were strongly connected to livelihood in BTH and their relative influence was 57.39% in total. In addition, annual precipitation (8.88%) played relatively important roles in wetland area. The relative influence of the other nine variables was less than 6% each, and the sum of their influences was 33.73%. 

## 4. Discussion

Given the widely recognized negative impacts of humans on wetlands, identification of the patterns of wetlands changes in a quantitative manner could substantially aid evidence-based policy making. The utility of this method has been confirmed by research conducted in mangrove forests in Southeast Asia [13]. The data used in the present study was objective and enabled a general insight into the transformation of wetlands and related human activities. The changes in the area of different wetlands from 2000 to 2015 were analyzed in different provinces and geographic areas, and patterns of change were presented in a clear way. Several implementable and effective actions could be recommended to protect natural wetlands.

### 4.1. Main Wetland Changes in Different Areas

For the whole BTH, there was 6264.07 km^2^ of wetlands in 2015, with 73.93% of this total being artificial wetlands. Wetlands distributed in different provinces or geographic areas showed different patterns. Most of the wetlands were located in Hebei and Tianjin provinces because of the large area of Hebei and the coastal location around the Bohai Rim [33]. Over the study period, natural wetland area decreased and artificial wetlands increased. The loss of natural wetlands would result in a loss of ecosystem services [34,35], such as groundwater recharge, flood control, recycling of organic waste, and wildlife habitats. Reservoirs/ponds was the dominant artificial wetland type and was mainly used by aquaculture, which will have serious negative consequences (e.g., water pollution and biodiversity loss) [36]. There is a need to consider the changing patterns of the various types of wetland and it is not sufficient to only consider the degeneration of wetlands through a reduction in area. Our study found that different wetland types in different areas changed accordingly. In general, land uses where there is a large level of involvement from human beings experience many changes. In Beijing, the dominant transformation was from artificial wetlands to natural wetlands, while the transformation tended to be the other way around in the other two provinces. The artificialization of wetlands was crucial in all three geographical areas.

### 4.2. The Drivers of Changes in Wetland Area

LULC changes: agriculture, artificialization, and grassland expansion were three dominant factors in the change of wetland area and all are closely linked to socioeconomic development in the BTH. The main drivers changed according to wetland types and locations. This phenomenon was closely related to the geographical location [37] and agreed with the main drivers of changes in wetland areas in other countries [4,13,23,38]. In BTH, the coastal areas were located around the Bohai Rim, which contains the most important port in the northern part of China. The plains in this research were part of the North China Plain, which is the major grain-producing area of China and is a location where many people make their living through agriculture [39]. Because of their productivity and ability to hold water, wetlands have become a priority in agricultural expansion. The global demand for animal protein is growing as the human population increases and diets transform in response to rising income and urbanization [40]. To satisfy this need, there has been an inevitable extension of food-related careers, especially aquaculture. The growth of the BTH is inhibited by the Taihang Mountains in the west and Yanshan mountains in the north. Grassland expansion has mainly occurred in Beijing, and is connected with water shortages. Unexpectedly, although urbanization is generally the most important factor in ecosystem decline [41], it did not directly impact on the decrease in the natural wetland area. This was because urbanization often occurs in a sequential conversion from natural to artificial wetlands and from artificial wetlands to built-up area.

**Figure 7:**
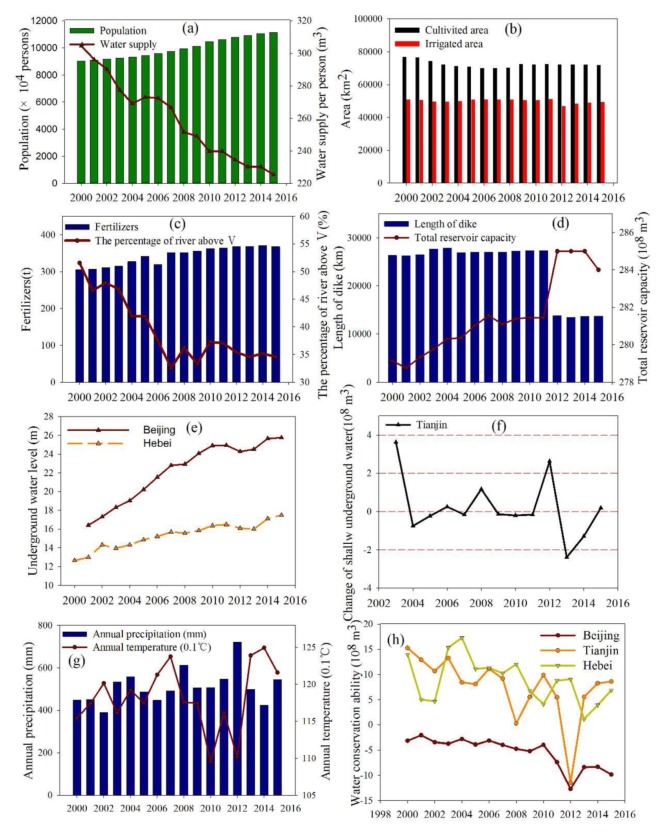
Changes in the environmental variables used in the Beijing-Tianjin-Hebei region (BTH) from 2000 to 2015. (**a**): population and water supply in BTH, (**b**):cultivated and irrigated area in BTH, (**c**): fertilizers and the percentage of river above grade V in BTH, (**d**): length of dikes and total reservoir capacity in BTH, (**e**): underground water level in Beijing and Hebei, (**f**): change of shallow groundwater in Tianjin, (**g**): annual precipitatiopn and temperature in BTH, (**h**): water conservation ability in Beijing, Tianjin and Hebei.

High density human activity: there were five types of high-density human activities that had impacts in this study. First, the population in the BTH continually increased from 2000 to 2015, and therefore more resources, especially land, water, and food, were required to meet the needs of society. The water supply per person decreased from 304.54 m^3^ in 2000 to 225.32 m^3^ in 2015 (Figure 7a). Second, the big area of cultivated and irrigated land has led to an overexploitation of the local water resources (Figure 7b). Third, there has been a worsening trend of water pollution, with the percentage of the river area in the BTH having above Grade Ⅴ water quality decreasing from 51.55% to 34.58% (Figure 7c). An increase in the area of cropland will increase the application of pesticides and fertilizers (Figure 7c), which will definitely accelerate non-point source pollution. Fourth, a large amount of reservoirs and dikes affect the hydrological environment and the connection between natural wetlands and the plains surrounding them. (Figure 7d). The rapid proliferation of such structures has caused the wide spread conversion and fragmentation of wetland habitats and a diminution of ecological flows [42,43]. Fifth, groundwater recession was a serious problem in all three provinces because of the over exploration (Figure 7e–f).

Effects of natural factors: previous studies have shown that precipitation and temperature determine wetland plant physiology [44], and changes in these parameters, especially temperature [45], would result in changes in wetland hydrology [46]. Figure 7g shows that the annual precipitation and temperature have slowly increased in the study area. The magnitude and direction of the hydrologic response would depend on whether climate change increased or decreased the water deficit and if the reaction due to climate change was aridity-specific [47]. Rising temperature will induce an increase in water temperature and there would be less dissolved oxygen, which could affect a number of animals [48]. Additionally, coastal wetlands are more sensitive to climate change [42]. The ability to conserve water, which can reflect the ability of ecosystems to contain water and can be calculated by an inflow minus outflow, displayed an obvious decreasing trend (Figure 7h). This may be connected to the extent of impervious surface area.

Effects of policies on wetlands: throughout our study period, the local governments established several policies related to wetlands. Legislation is a necessary way to protect wetlands, with the Beijing Wetland Protection Regulations, implemented in 2013, being a good example. Development plans established by governments have a huge influence on LULC changes [36]. Binhai New Area in Tianjin was designated to be a principle development area in the Urban Master Plan of Tianjin (2005–2020). The Beijing Wetland Protection Project Plan from 2000 to 2010 was established to protect wetlands in Beijing, while the Capital Water Resources Sustainable Utilization Plan in the Early 21st Century from 2001 to 2005 and the Grain to Green project in Hebei were important strategies devised to guarantee water supply to Beijing. Wetland recovery was an important aspect of the Red line plan of ecological land protection in Tianjin and was published in 2014. Water regulations, such as the south-to-north water transfer project, reclaimed water project, and sewage treatment and water pricing adjustments, also had impacts on wetlands. These actions can have a profound direct or indirect influence on wetlands.

### 4.3. Evidence-Based Policy Advice

The BTH has established a goal of accomplishing 340 km^2^ of wetlands recovery by 2020. A focus on natural wetland recovery and protection is recommended because of the primary role it plays in providing ecosystem services. Additionally, policies should be made according to the drivers in specific locations. The results presented here indicate that using one single wetland conservation policy throughout a region would be inadequate. A smart policy for wetland conservation requires these differences to be acknowledged throughout the territory and should propose different actions for different locations. 

This study recommended that provinces act as basic administrative units to offer advice according to specific drivers. For Beijing, grassland expansion was the dominant driver and this factor has a strong relationship with water shortages. This problem could be best solved in two ways. Within Beijing, policies to improve the utilization of water resources and restrict groundwater exploitation should be implemented, and the use of rainfall and wastewater should also be improved. Outside Beijing, the government should take effective actions to guarantee the inflow of water, using the grain to green and south-to-north water transfer projects as examples. For Hebei, agricultural production was the dominant driver, and therefore adjustment of the agricultural structure and the realization of agro-technology should be prioritized to limit the expansion of cropland. Grassland expansion also requires attention, while improvements in water-saving irrigation technology, water price regulation, and pollution treatment should also be taken into consideration. For Tianjin, applications to build reservoirs/ponds and land reclamation should be carefully and critically scrutinized. At the same time, the government should develop the sustainable intensification of aquaculture to realize production by using fewer inputs. In particular, there is an urgent need to develop a sustainable intense aquaculture model. For example, shrimp farming is a more water-demanding system than pangasius farming [40]. Under the water stressed conditions in the BTH, the government should, therefore, encourage pangasius farming rather than shrimp farming. Even in the same province, different levels of protection should be given to different geographic areas, especially the coastal areas in Heibei and Tianjin. Deficit irrigation deserves more attention for a range of different crops and different environments to make full use of water resources [49], especially after the Chinese Government’s proposed production increase of 50 billion kg of grain between 2015 and 2020. It is also imperative to provide awareness about wetland losses to the local people through educational programs in all three provinces. It is important to be aware that wetlands have many times greater potential to improve economic turnover than agricultural or aquacultural land [50].

### 4.4. Future Research

In this study, patterns of change were analyzed based on the ways in which wetland areas were converted to other land uses. President Xi launched a mission to overcome poverty by 2020 in the fifth plenary meeting of the 18th Central Committee in 2015. Agriculture and aquaculture, as primary livelihoods, are likely to increase in activity, which will place more pressure on wetlands. Master plans for these three provinces will focus on the development of the northwest and coastal areas [51,52,53]. Many changes can be expected in wetlands in the mountainous and coastal areas over the next few decades. Biodiversity and ecosystem functions are likely to show nonlinear responses to increasing land use intensification, and management alternatives with limited ecological losses that still satisfy economic gains might exist [54,55]. Under these political circumstances, future studies will examine the relationships between income, biodiversity, and ecosystem services during wetland conversation.

## Figures and Tables

**Table 1:** Wetland area in the Beijing-Tianjin-Hebei Region (BTH) from 2000 to 2015.

Wetland Types	2000		2015		Changes from 2000 to 2015	
	Area/km^2^	Percent/%	Area/km^2^	Percent/%	Area */km^2^	Percent/%
Herbaceous	985.54	14.81	826.67	13.20	−158.87 *	−16.12
Lake	22.72	0.34	35.20	0.56	12.48	54.96
Reservoir/pond	4298.07	64.58	4447.09	70.99	149.01	3.47
Rivers	1109.40	16.67	771.22	12.31	−338.18 *	−30.48
Canal/Channel	239.75	3.60	183.90	2.94	−55.85 *	−23.30
Total	6655.49	100.00	6264.07	100.00	−391.42 *	−5.88

Note: * minus signal (−) means wetland area decreased from 2000 to 2015.

**Table 2:** Contribution of different driving forces calculated as the percentage change between 2000 and 2015 in in different geographic areas in Beijing-Tianjin-Hebei region (BTH).

		Changing Patterns /%					
Landforms	Wetland Type	Urbanization	Agriculture	Artificialize	Internal Conversion	Grassland Expansion	Other
Coastal area	Herbaceous swamp	3.48	3.06	82.44	7.13	0.22	3.67
	Lakes	-	-	-	-	-	-
	Rivers	12.84	25.66	44.24	3.61	9.34	4.31
	Total	8.00	13.99	63.97	5.43	4.63	3.98
Plain	Herbaceous swamp	3.72	13.00	66.51	5.57	8.98	2.22
	Lakes	0.31	7.98	79.58	0.16	0.20	11.76
	Rivers	5.57	45.44	6.47	1.27	30.10	11.15
	Total	4.78	31.56	32.16	3.10	21.05	7.35
Mountainous area	Herbaceous swamp	1.43	46.12	21.92	2.13	16.11	12.30
	Lakes	15.35	5.20	0.00	6.92	27.83	44.70
	Rivers	5.70	43.52	0.99	0.44	40.41	8.95
	Total	4.33	44.27	7.81	1.00	32.46	10.12
In Total	Herbaceous swamp	3.42	16.89	61.35	5.19	9.60	3.56
	Lakes	4.13	7.23	58.88	1.88	7.23	20.65
	Rivers	5.75	44.66	6.28	1.16	31.55	10.59
	Total	4.79	33.16	29.06	2.82	22.46	7.70

**Table 3:** Contribution of different driving forces calculated as the percentage change between 2000 and 2015 in different provinces in Beijing-Tianjin-Hebei region (BTH).

		Changing Patterns/%					
Province	Wetland Type	Urbanization	Agriculture	Artificialize	Internal Conversion	Grassland Expansion	Other
Beijing	Herbaceous swamp	13.25	8.62	20.41	3.15	43.95	10.61
	Lakes	0.31	7.98	79.58	0.16	0.20	11.76
	Rivers	5.92	10.51	7.06	1.43	55.60	19.48
	Total	7.35	10.10	10.59	1.77	52.61	17.58
Hebei	Herbaceous swamp	3.16	24.95	53.22	2.20	12.37	4.10
	Lakes	15.00	5.09	0.00	6.77	27.20	45.94
	Rivers	5.45	50.65	5.52	0.71	28.38	9.29
	Total	4.78	43.01	19.65	1.16	23.63	7.77
Tianjin	Herbaceous swamp	2.62	8.59	75.17	8.83	2.63	2.15
	Lakes	-	-	-	-	-	-
	Rivers	17.28	17.75	32.71	18.15	7.36	6.75
	Total	3.53	9.15	72.55	9.41	2.92	2.44
In Total	Herbaceous swamp	3.42	16.89	61.35	5.19	9.60	3.56
	Lakes	4.13	7.23	58.88	1.88	7.23	20.65
	Rivers	5.75	44.66	6.28	1.16	31.55	10.59
	Total	4.79	33.16	29.06	2.82	22.46	7.70
